# Multiple cutaneous metastasis of synchronous urothelial carcinoma of the bladder and the renal pelvis: a case report

**DOI:** 10.1186/s13256-019-1997-8

**Published:** 2019-02-14

**Authors:** M. Ghalleb, M. A. Ayadi, S. Slim, I. Zemni, R. Doghri, J. Ben Hassouna, K. Rahal

**Affiliations:** 1 Surgical Oncology Department, Institute Salah Azaiez de Cancer, Tunis, Tunisia; 2 Pathology Department, Institute Salah Azaiez de Cancer, Tunis, Tunisia; 3 Faculté de Medicine Tunis El Manar, Tunis, Tunisia

**Keywords:** Bladder, Urothelium, Carcinoma, Metastasis, Skin

## Abstract

**Introduction:**

Cutaneous metastatic disease arising from urinary tract carcinoma is rare and associated with a poor prognosis. We report a case of metastatic disease occurring in a patient treated for synchronous urothelial tumor of the bladder and left renal pelvis.

**Case presentation:**

A 61-year-old Caucasian man was treated for a synchronous urothelial tumor of the bladder and left renal pelvis. He had an *en bloc* radical cystectomy and left ureteronehprectomy associated with a cutaneous transileal urinary diversion and lymph node dissection. He was scheduled for chemotherapy but was lost to follow-up. He consulted 1 year later with growing skin tumors that were confirmed to be metastatic disease, and he was referred to the oncology department for palliative chemotherapy.

**Conclusion:**

Cutaneous metastatic disease is a rare entity with poor prognosis. The main treatment remains chemotherapy; however, single-site metastasis should be considered for metastasectomy.

## Introduction

Cutaneous metastatic disease (CMD) arising from primary visceral carcinoma is rare, occurring with an overall incidence of 0.3% to 5.3% [1, 2]. Urologic cancers appear less likely than other primary malignancies to metastasize to the skin, with an incidence of 0.73% [2]. We report a case of multiple-site skin metastasis in a patient followed for synchronous urothelial carcinoma of the bladder and the left renal pelvis. This work is reported using the care checklist guidelines.

## Case presentation

A 61-year-old Caucasian man with no past medical history presented to another teaching hospital with a 2-week history of hematuria. He had a transurethral resection of a 3-cm papillary bladder tumor located near the left ureter meatus. The final histologic examination led to the conclusion that it was a urothelial carcinoma pT2 G3.

The patient was referred to our outpatient clinic after 2 months. He still reported hematuria. The result of his physical examination was totally normal. All the biological workup was normal except for a decreased hemoglobin level (10 g/dl). Thoracoabdominopelvic computed tomography (CT) showed a 4-cm heterogenic and enhanced bladder tumor with invasion of the left ureter and another 3-cm mass with the same characteristic located in the left renal pelvis. No other sign of malignant disease was found by CT.

The multidisciplinary team decided to start with upfront surgery. The patient had a midline laparotomy, which revealed that the abdominal cavity was free of ascites and calcinosis. The liver was free of disease. Therefore, the patient had an *en bloc* radical cystectomy and a left ureteronephrectomy associated with para-aortic and bilateral pelvic lymph node dissection. He also had a cutaneous transileal urinary diversion. The surgery lasted 245 minutes with no need for blood transfusion.

The immediate follow-up was normal. The patient was discharged 1 week after surgery.

The final histologic examination showed a synchronous high-grade urothelial carcinoma of the bladder (pT3) and the left renal pelvis (pT3) with free margin.

All the lymph nodes dissected were free of disease: nine para-aortic lymph nodes, five right pelvic dissection lymph nodes, and seven left pelvic lymph nodes.

The multidisciplinary team decided to add adjuvant chemotherapy. However, the patient was lost to follow-up.

**Fig. 1:**
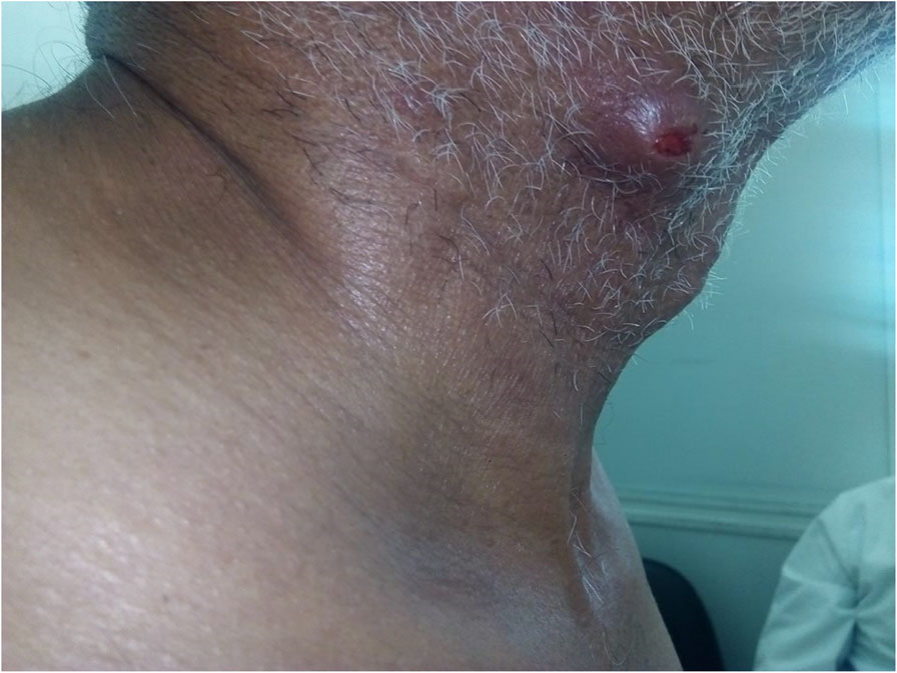
Cervical cutaneous metastasis of urothelial bladder carcinoma in our 61-year-old patient

**Fig. 2:**
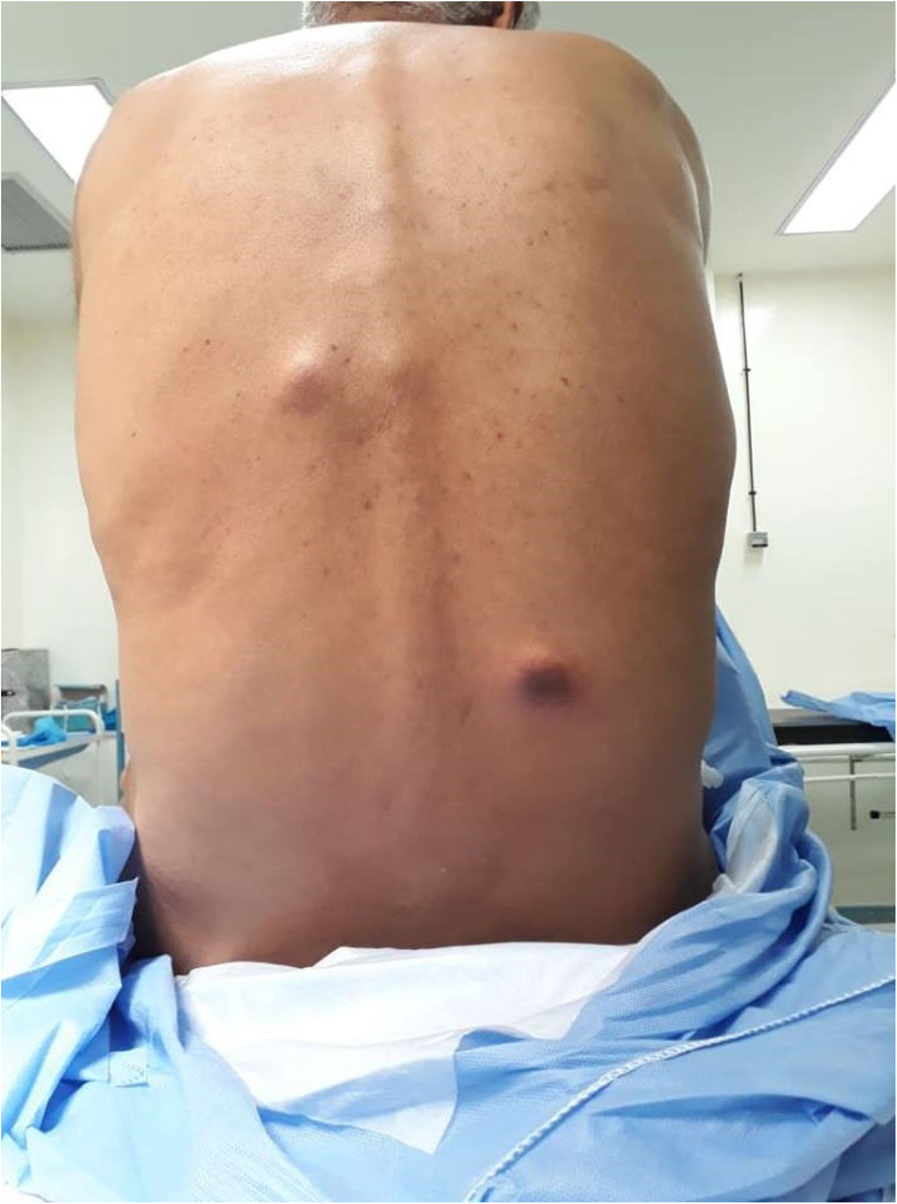
Back cutaneous metastasis of urothelial bladder carcinoma in our 61-year-old patient

**Fig. 3:**
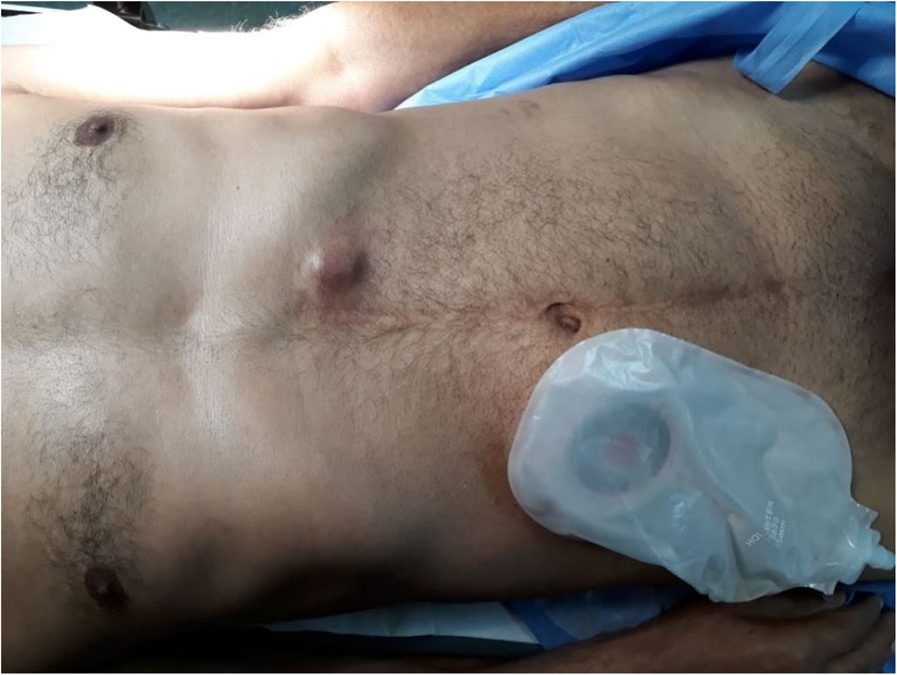
Left hypochondriac metastasis of urothelial bladder carcinoma in our 61-year-old patient

He consulted our outpatient clinic after 1 year for cutaneous masses located in the left hypochondriac (1), the back (2), and the cervical region (1) (Figs. 1, 2 and 3). No other abnormal signs were found in the physical examination.

Thoracoabdominopelvic CT was performed, which showed multiple pulmonary metastases but no sign of local recurrence.

**Fig. 4:**
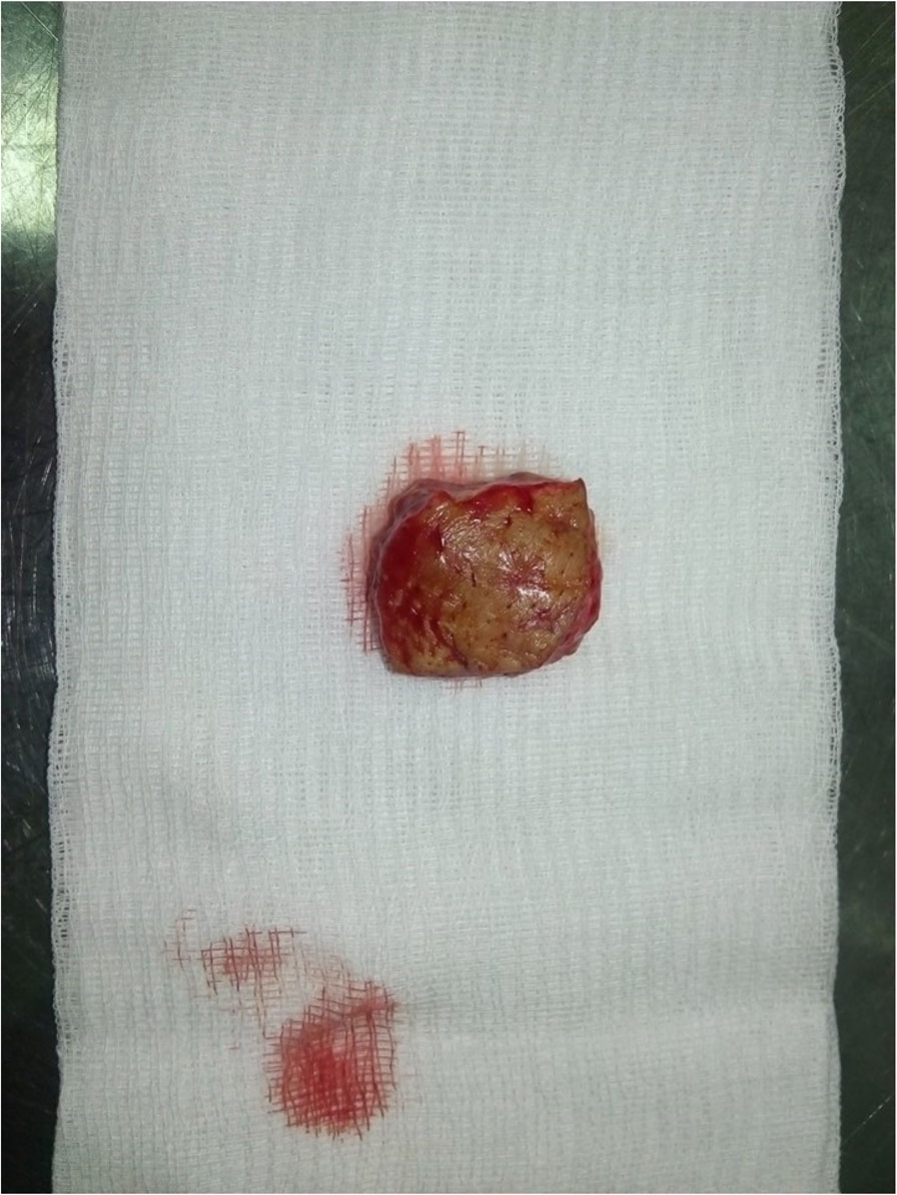
Incisional biopsy of the left hyponchondriac metastasis

**Fig. 5:**
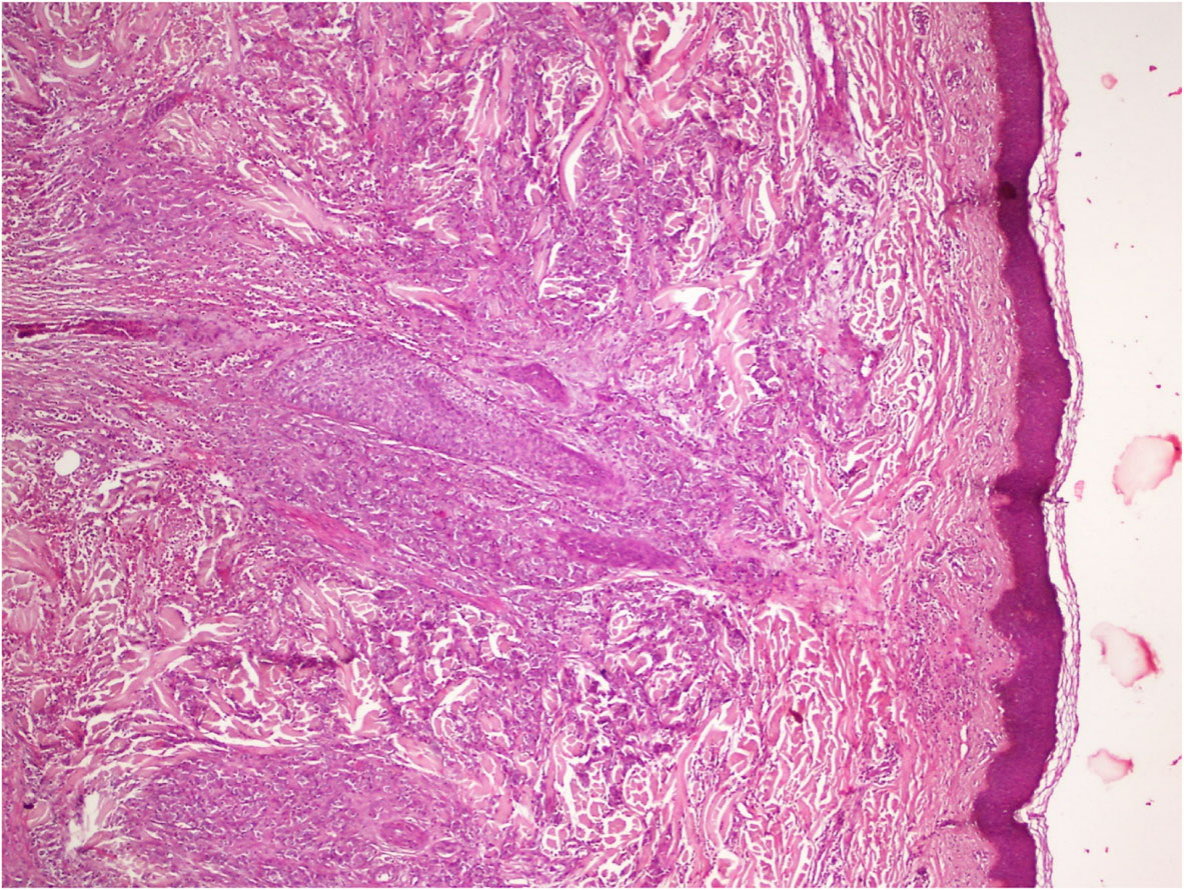
H&E coloration showing the tumoral proliferation under the skin layer

**Fig. 6:**
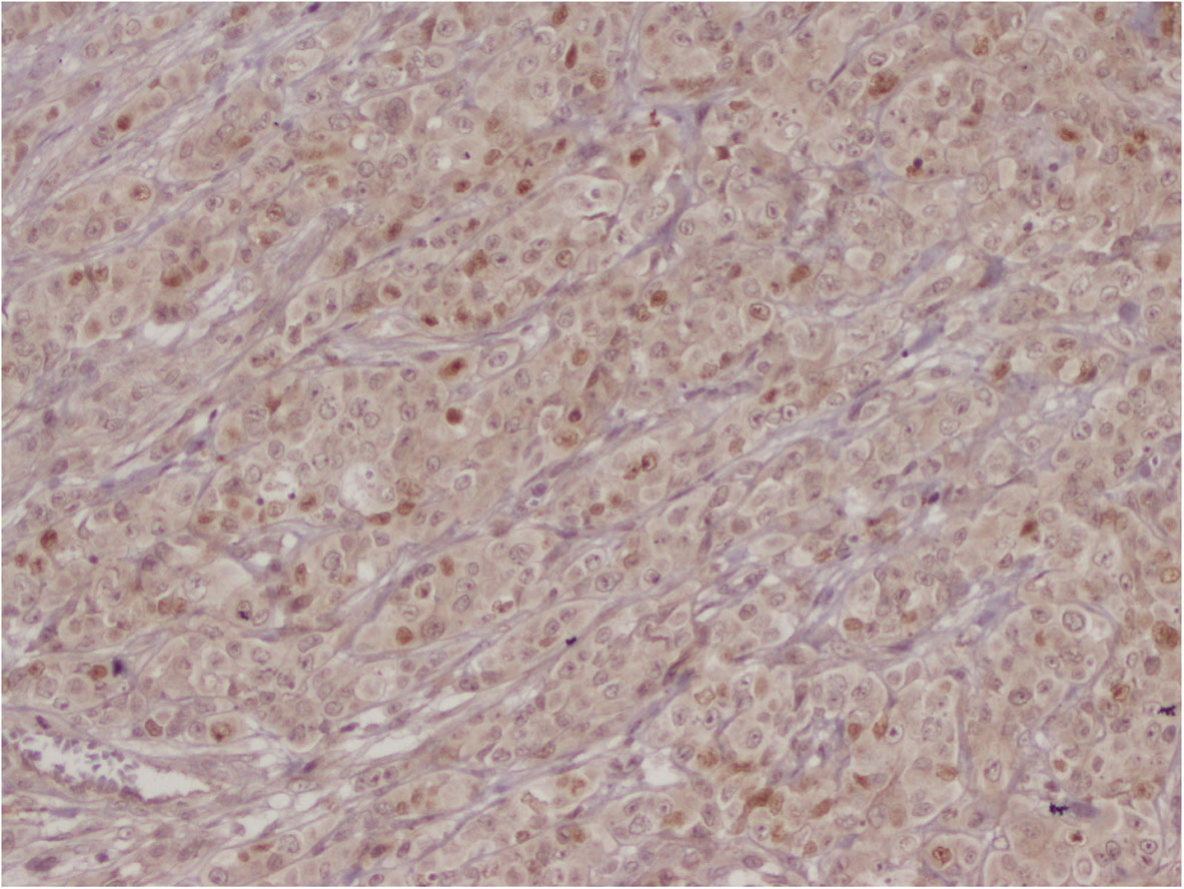
IHC positive for P63

**Fig. 7:**
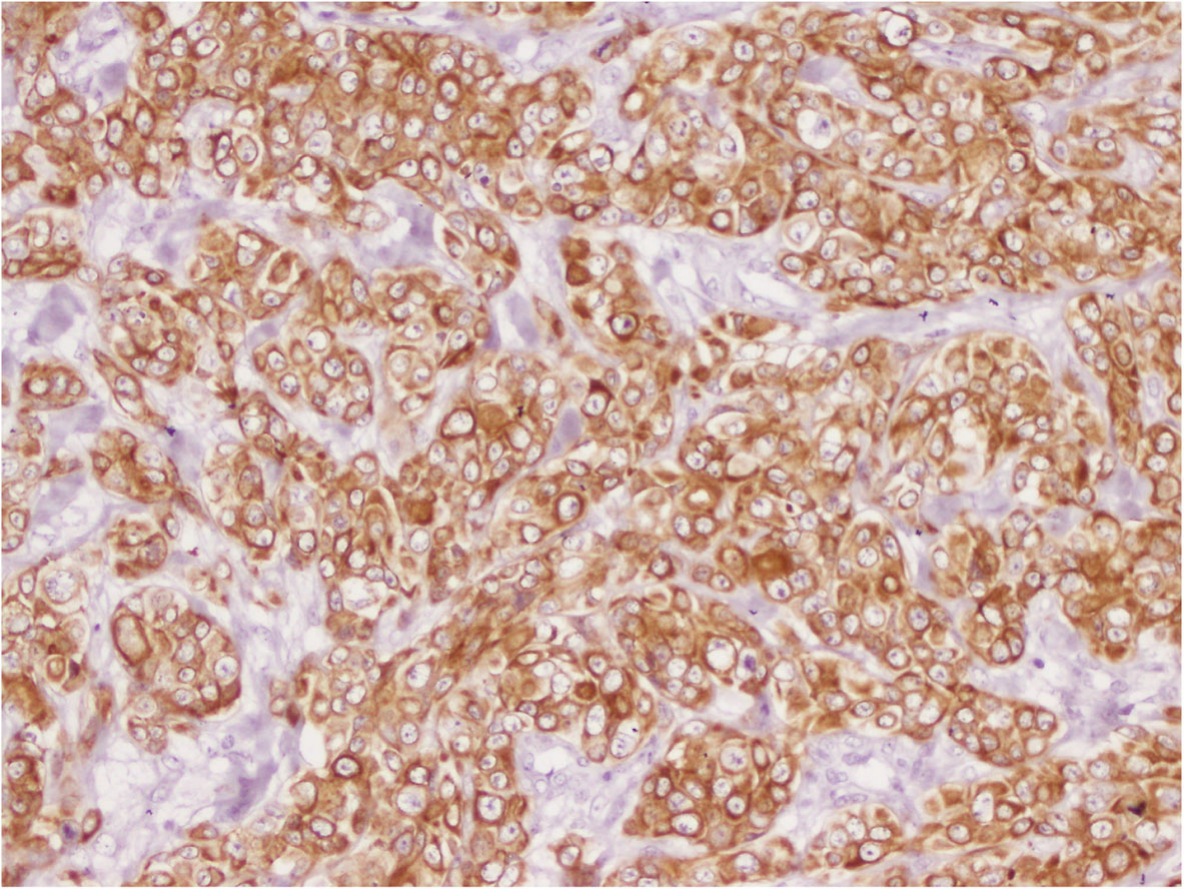
IHC positive for CK7+

The patient had a biopsy of the left hypochondriac lesion (Fig. 4), and the histology confirmed the metastatic origin. The tumor was CK7- and P63-positive (Figs. 5, 6 and 7).

The patient was then referred for chemotherapy. However, he never received treatment because of deteriorating general status.

The patient died 1 month after the biopsy.

## Discussion

CMD is an uncommon occurring for urinary tract carcinoma. The incidence of urinary malignancies metastasizing to the skin varies from 0.73% to 3.8% [1, 2].

According to a meta-analysis of seven studies with a total number 22,297 patients performed by Krathen *et al.* [3], the overall incidence of visceral tumors metastasizing to the skin is 5.3%. Krathen *et al.* also reported similar rates between renal and bladder cancer, ranging from 3.4% to 4%.

In the literature, there are four different mechanisms of skin invasion reported to be associated with the disease [4]:

1. Direct invasion from underlying tumor
2. Operative scar metastasis
3. Lymphatic spread
4. Hematogenous spread

In our patient, hematogenous spread seemed to be the most probable mechanism, owing to the absence of lymph node involvement in the first surgery, the presence of pulmonary metastasis, the absence of locoregional relapse, and the absence of operative scar metastasis.

In the majority of cases, skin metastases develop in the locoregional skin through lymphatic spread [5]. Few cases such as ours have CMD in a location far from the primary tumor [5].

Cutaneous metastasis is rare and generally accepted as the late manifestation of systemic spread [6].

Some authors suggest that CMD from the urinary tract is not rare but is usually overlooked and less frequently reported [2]. CMD can appear as a benign lesion, and diagnosis can be misled [1]. Therefore, it is recommended to biopsy every suspicious lesion [2].

Concerning histology, cutaneous metastases predominantly involve the dermis, and there is usually a narrow zone of superficial dermis separating the lesion from the epidermis [2]. That is why some authors recommend excisional or punch biopsy rather than superficial shaving biopsy [2].

As stated above, CMD is usually associated with other metastatic sites; therefore, the preferred treatment is chemotherapy [7].

The European Association of Urology recommends offering chemotherapy as the first option in cases of distant recurrence and to consider metastasectomy in cases of a unique metastasis site [7]. CMD is usually associated with a poor prognosis with survival less than 12 months [4].

## Conclusion

CMD from urinary tract tumors is a rare and underreported presentation associated with poor prognosis. It is usually associated with other more common metastatic sites. The excisional biopsy or punch biopsy is preferred to shave biopsy for the histological diagnosis. Chemotherapy is the first treatment option; however, metastasectomy can be discussed in cases with unique metastasis sites.
